# Prevalence of anti-nuclear antibodies in COVID-19 patients: a remarkable 2.5-fold rise compared to healthy controls

**DOI:** 10.1007/s11845-025-03886-8

**Published:** 2025-02-01

**Authors:** Faruk Dişli, Sedat Yıldız

**Affiliations:** 1 Faculty of Physiotherapy and Rehabilitation Department of Basic Physiotherapy, Bingöl University, Bingöl, Türkiye; 2 Faculty of Medicine, Department of Physiology, İnönü University, Malatya, Türkiye

**Keywords:** ANA, Autoantibody, Coronavirus, COVID-19, SARS-CoV-2

## Abstract

**Background:**

The SARS-CoV-2 virus, responsible for COVID-19, has the potential to induce the formation of antibodies that target self-antigens. This study aimed to investigate the prevalence of antinuclear antibodies (ANA), the most common type of autoantibody, in both healthy individuals and those recovered from COVID-19.

**Methods:**

This retrospective study involved 400 participants (200 with COVID-19 and 200 healthy controls), aged 18 to 60 years, without any chronic diseases, including autoimmune conditions. Analyses were performed using three different ANA ELISA test kits (anti-dsDNA, anti-ENA, and anti-Hep-2 nucleus tests) that were developed and validated.

**Results:**

Among the 200 healthy individuals, 38 (19%) tested positive for dsDNA, 37 (18.5%) for ENA, and 30 (15%) for Hep-2 nucleus antibodies. The rates of ANA positivity were significantly higher in individuals with COVID-19, with 97 (48.5%) positive for dsDNA, 81 (40.5%) for ENA, and 84 (42%) for Hep-2 nucleus antibodies (*p* < 0.05).

**Conclusion:**

One in every two to three individuals with COVID-19 exhibited ANA positivity, indicating that the SARS-CoV-2 virus poses a significant risk regarding autoimmunity. Furthermore, the incidence of ANA in healthy individuals was observed to be higher than the literature average.

## Introduction

The severe acute respiratory syndrome coronavirus 2 (SARS-CoV-2), responsible for coronavirus disease (COVID-19), not only causes a variety of symptoms but also has the potential to initiate the production of autoantibodies that target self-antigens [1, 2]. Studies have indicated that individuals recovering from COVID-19 may exhibit elevated levels of autoantibodies, raising concerns about potential long-term health effects [3–5]. This phenomenon may manifest as autoimmune responses affecting organs beyond the respiratory system, such as the heart, kidneys, and brain, in certain individuals [6]. Therefore, it is essential to assess how the prevalence of these autoantibodies, which represent a significant risk factor for autoimmunity, has changed in conjunction with the COVID-19 pandemic.

Among autoantibodies, the most common are antinuclear antibodies (ANA) [7]. ANA, which can lead to widespread inflammation and tissue damage in autoimmune diseases, has been considered one of the factors associated with poor prognosis in COVID-19 cases [8]. Notably, elevated levels of ANA have been observed more frequently in patients who experienced severe COVID-19, and this has been linked to reduced treatment efficacy and delayed recovery [8, 9]. Additionally, it has been suggested that ANA may be associated with tissue damage observed in individuals who have recovered from COVID-19.

Autoantibodies pose a potential risk for various autoimmune diseases. Given the ability of the SARS-CoV-2 virus to induce the production of autoantibodies, public health concerns may increasingly shift toward autoimmunity. It is crucial to determine the prevalence of these autoantibodies in individuals who have recovered from COVID-19 across all countries to identify populations at risk for autoimmunity. Although ANA can also be observed in healthy individuals (5–15%), a high prevalence of autoantibodies serves as a significant indicator that the population may be at risk for autoimmune disorders [10, 11]. The aim of our study is to compare the frequency of ANAs in individuals who have recovered from COVID-19 with that in healthy individuals who have not contracted the virus. The data obtained from this study will elucidate risk rates in Türkiye and facilitate the comparison of inter-country risk rates through similar screenings conducted in other nations.

## Methods

Prior to the study, ethical approval was obtained from the Inonu University Health Sciences Non-Interventional Clinical Research Ethics Committee (2022/557). The study was designed retrospectively, utilizing serum samples collected during the pandemic period. Analyses were conducted at the Immunoassay Development Laboratory of the Inonu University Medical Faculty Physiology Department.

### Study design

**Fig. 1:**
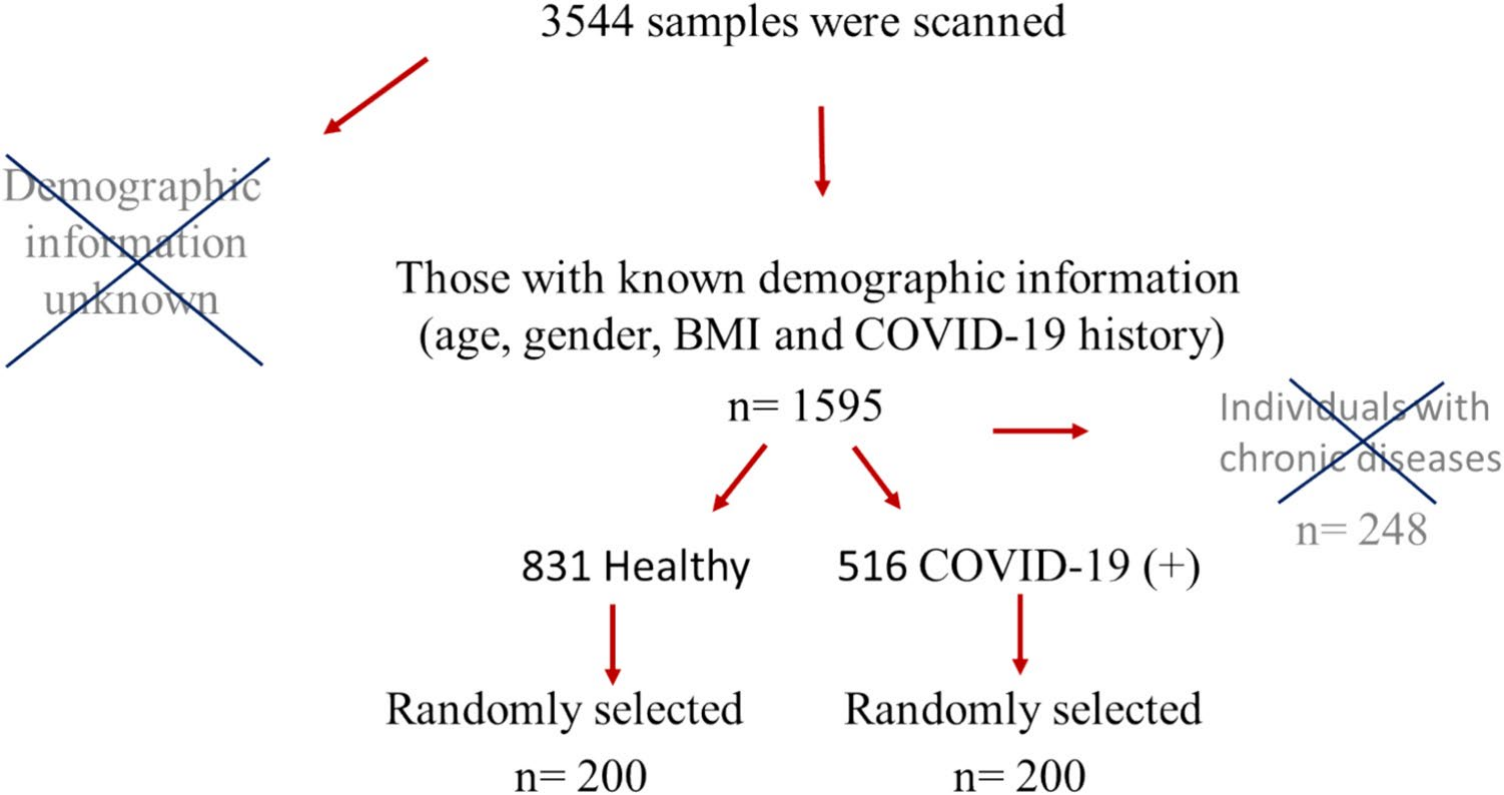
Determination of research samples. In the random selection process, samples were numbered and transferred to excel. The "RANDARRAY" function in excel was utilized to select 200 samples from both COVID-19 positive individuals and healthy controls

In this study, serum samples were obtained from individuals aged 18 to 60 who did not have any chronic diseases. Samples from individuals with chronic diseases or a history of autoimmune diseases were excluded from the study. To determine the prevalence of ANA following COVID-19, the population of Malatya province (approximately 800,000) was considered, and the required sample size was calculated to be *n* = 400. A total of 3,544 serum samples were collected during the pandemic period (between 2019 and 2022). Demographic information was unavailable for 1,949 samples, which were excluded from the study. Of the remaining 1,595 serum samples, those from individuals with chronic diseases such as asthma, diabetes, and autoimmune conditions were removed (*n* = 248). This left 1,347 samples, which were categorized into two groups: individuals who had contracted COVID-19 (*n* = 516) and healthy individuals who had not (*n* = 831). From each group, 200 samples were randomly selected using Excel’s random sampling function to create the experimental groups (Fig. 1). Thus, a total of 200 healthy individuals (100 men, 100 women) and 200 COVID-19 recovered individuals (80 men, 120 women) were included in the study.

The criteria for COVID-19 diagnosis required the presence of at least one symptom (such as loss of taste, loss of smell, fever, etc.) and a positive PCR test and/or elevated SARS-CoV-2 antibody levels. Individuals who did not meet the diagnostic criteria established by the Ministry of Health and had negative antibody tests were classified as not having COVID-19.

The samples were analyzed using the ELISA method, and three distinct ELISA test kits were developed and validated for the measurement of ANA. Two of these tests, anti-dsDNA and anti-ENA, are widely utilized in both clinical practice and research literature. The third test utilized intact cell nuclei as the antigen. The Hep-2 cell line, commonly used in immunofluorescence assay (IFA) techniques and rich in ANA antigens, was selected as the source of nuclei for this test. By employing the Hep-2 nuclei, which contain various ANA antigens beyond dsDNA and ENA, we aimed to ensure that no positive ANA samples were overlooked.

### Development of ANA ELISA test kits

The calf thymus gland was utilized as the antigenic source for the dsDNA and ENA tests, while nuclei were isolated from the Hep-2 cell line. All three test kits were designed according to the indirect ELISA method. However, there were differences in the attachment of antigens to the well bottom. In the ENA test, the antigens were directly attached to the bottom of the wells in PBS, while in the dsDNA test, protamine sulfate was employed as a linker substance to bind DNA to the wells. In the Hep-2 nucleus test, nuclei were bound to the bottom using a commercial solution (Kementec, WellChampion) and subsequently blocked with this solution. 1% BSA was used for blocking in the dsDNA and ENA tests. Additionally, unlike the other two tests, in the Hep-2 nucleus test, Triton X was used to enhance membrane permeability, as intact nuclei were utilized. All subsequent steps were similar for all three tests. Serum samples were diluted 1/100 and added to the wells alongside negative and positive control samples. Subsequently, anti-human IgG conjugated with biotin and streptavidin peroxidase was added. Plates were washed three times with 0.05% Tween before each solution was added. Finally, a chromogenic substrate (tetramethylbenzidine, TMB) was added, and the reaction was halted with 11% H₂SO₄. The plates were then read using a spectrophotometer at 450 nm.

### Validation of test kits: intra-assay and inter-assay data

The intra-assay (within-test) and inter-assay (between-test) CV percentages were obtained by performing repeated measurements of two negative and two positive samples across four different plates. These values were computed using the formula: “% CV = (OD standard deviation / OD mean) × 100.” The intra- and inter-assay means for the dsDNA, ENA, and Hep-2 nucleus tests were 7.8%, 7.5%, and 9.9%, respectively.

### Sensitivity and specificity analysis

For the sensitivity and specificity analyses, dsDNA and ENA positive and negative samples were analyzed at the İnönü University Medical Faculty Central Microbiology Laboratory (DIESSE Diagnostica Senese S.p.A. Chorus ds-DNA-G, ANA-8). For the dsDNA test, 48 positive and 48 negative samples were used, while for the ENA and Hep-2 tests, 12 negative and 12 positive samples were used. The positive samples used in the Hep-2 nucleus test were positive for both dsDNA and ENA. The sensitivities for the dsDNA, ENA, and Hep-2 nucleus tests were found to be 93.8%, 83.3%, and 90%, respectively, while the specificities for these tests were 91.7%, 83.3%, and 87.5%, respectively.

### Cross-reactivity with different types of autoantibodies

Serum samples that exhibited negative ANA levels but tested positive for various autoantibodies (anti-cyclic citrullinated peptide (anti-CCP) [*n* = 2], anti-β2-glycoprotein I [*n* = 2], anti-myeloperoxidase (anti-MPO) [*n* = 1], anti-proteinase 3 (anti-PR3) [*n* = 2], and anti-cardiolipin [*n* = 1]) were analyzed to assess cross-reactivity in dsDNA, ENA, and Hep-2 nucleus testing. The results indicated that the five different autoantibodies examined did not exhibit cross-reactivity in the dsDNA and Hep-2 nucleus tests; however, anti-CCP and anti-β2-glycoprotein demonstrated low levels of cross-reactivity in the ENA test.

### Sample analysis

While the majority of the samples analyzed consisted of serum, a few samples were derived from plasma, and some were hemolyzed serum. Therefore, the impact of serum, plasma, and hemolyzed serum samples from the same individual on the test results was evaluated. The findings indicated that samples from all three types did not significantly affect the test outcomes.

ANA measurements were conducted using three validated ELISA test kits (dsDNA, ENA, and Hep-2 nucleus). The cut-off value was established based on the negative control optical density (OD) multiplied by 1.5, as determined in the validation studies. The obtained OD values were converted to an antibody index using the formula: Ab index = Sample OD / Cut-off OD. Samples with an index value of < 1.0 were classified as ANA IgG negative, while those with an index value > 1.0 were classified as ANA IgG positive. The test was considered valid if the antibody index of the positive control was > 1.1 and that of the negative control was < 0.9.

## Results

### Demographic information of participants

The study included individuals who had not previously received a COVID-19 vaccine. The mean age of the healthy participants was 40.1 ± 7.4 years, while the mean age of the COVID-19 group was 38.2 ± 7.8 years. The duration since the onset of COVID-19 was 83.9 ± 50.3 days.

### Long-term storage of samples did not affect antibody levels

Since the study was planned retrospectively and utilized serum samples stored at −20 °C for 2–3 years, it was essential to assess whether the antibodies had deteriorated during this period. When these samples were initially collected, anti-SARS-CoV-2 IgG levels were measured and documented. From these samples, 4 positive and 4 negative samples were selected, and the anti-SARS-CoV-2 IgG levels were re-measured using the same kit (Quantitative CORonavirus Antibody ELISA Test, Yimmunek A.S., Malatya, Turkey). The results of the measurements were found to be consistent with the initial measurements (Initial Measurement**:** 0.99 ± 0.14 OD.

Final Measurement**:** 0.98 ± 0.23 OD (*p* > 0.05), leading to the conclusion that other antibodies, such as ANA, could also be reliably measured.

### Prevalence of ANA in individuals with COVID-19 and healthy individuals

**Fig. 2:**
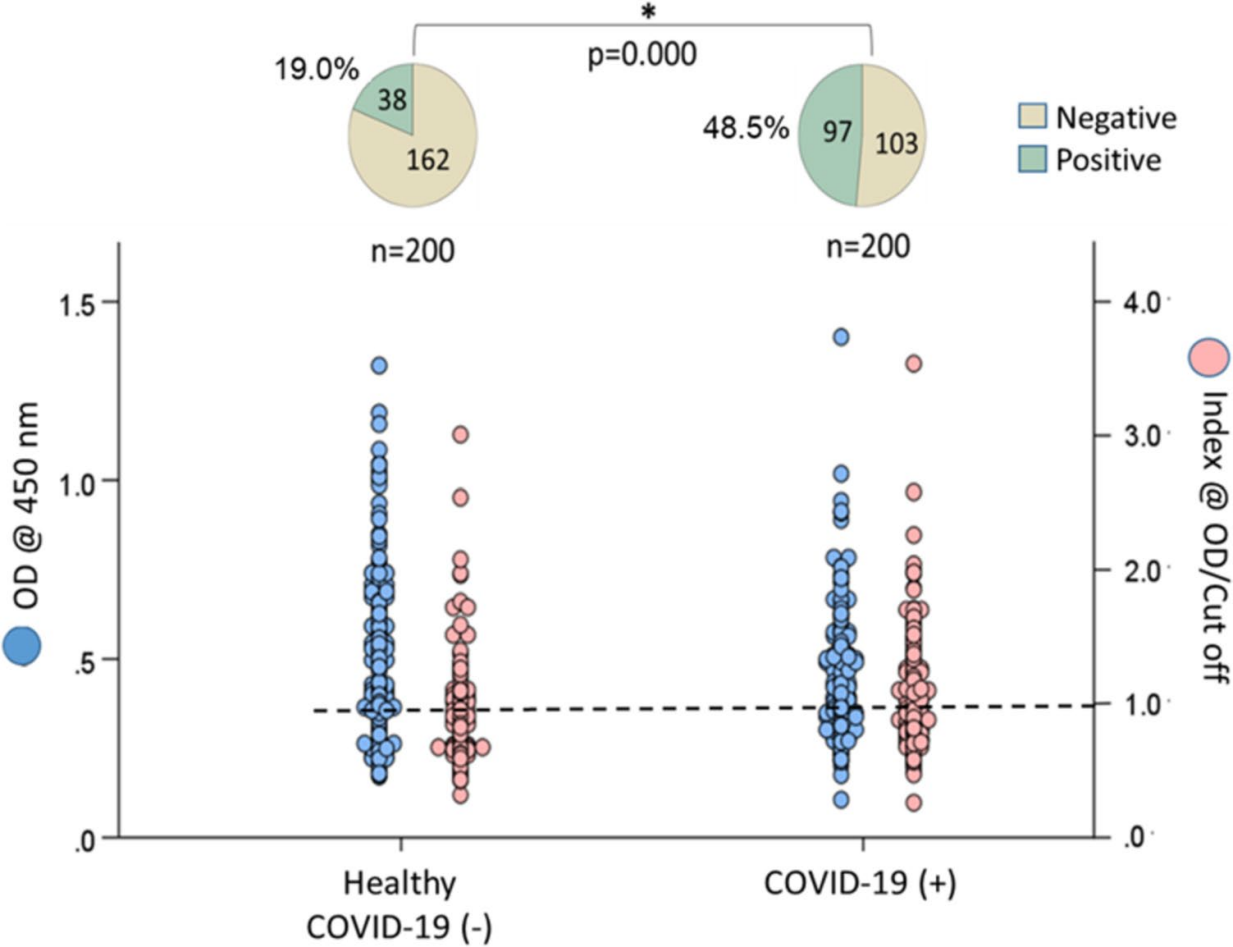
The rates of anti-dsDNA antibody positivity in individuals who have recovered from COVID-19 compared to healthy controls

**Fig. 3:**
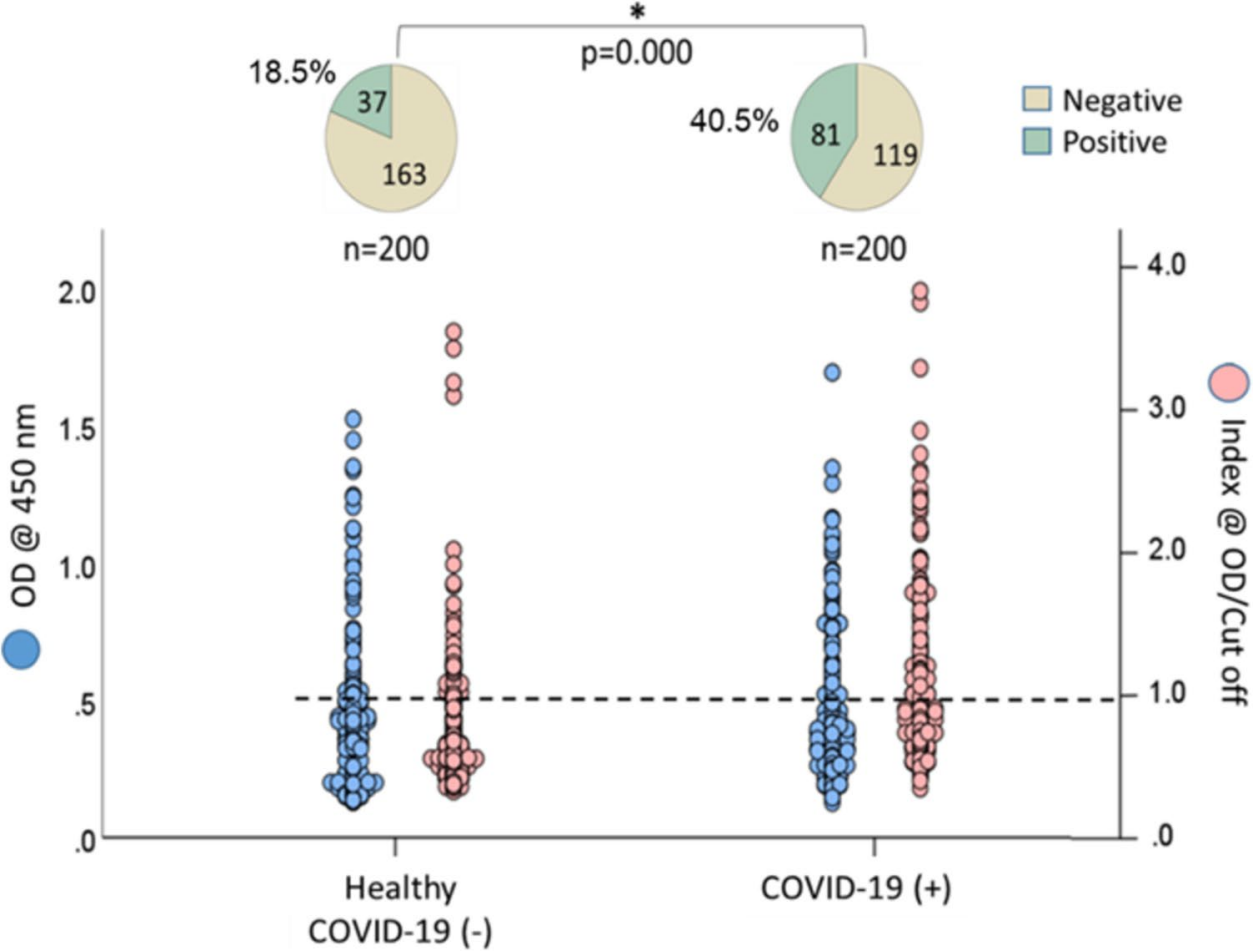
The rates of anti-ENA antibody positivity in individuals who have recovered from COVID-19 compared to healthy controls

**Fig. 4:**
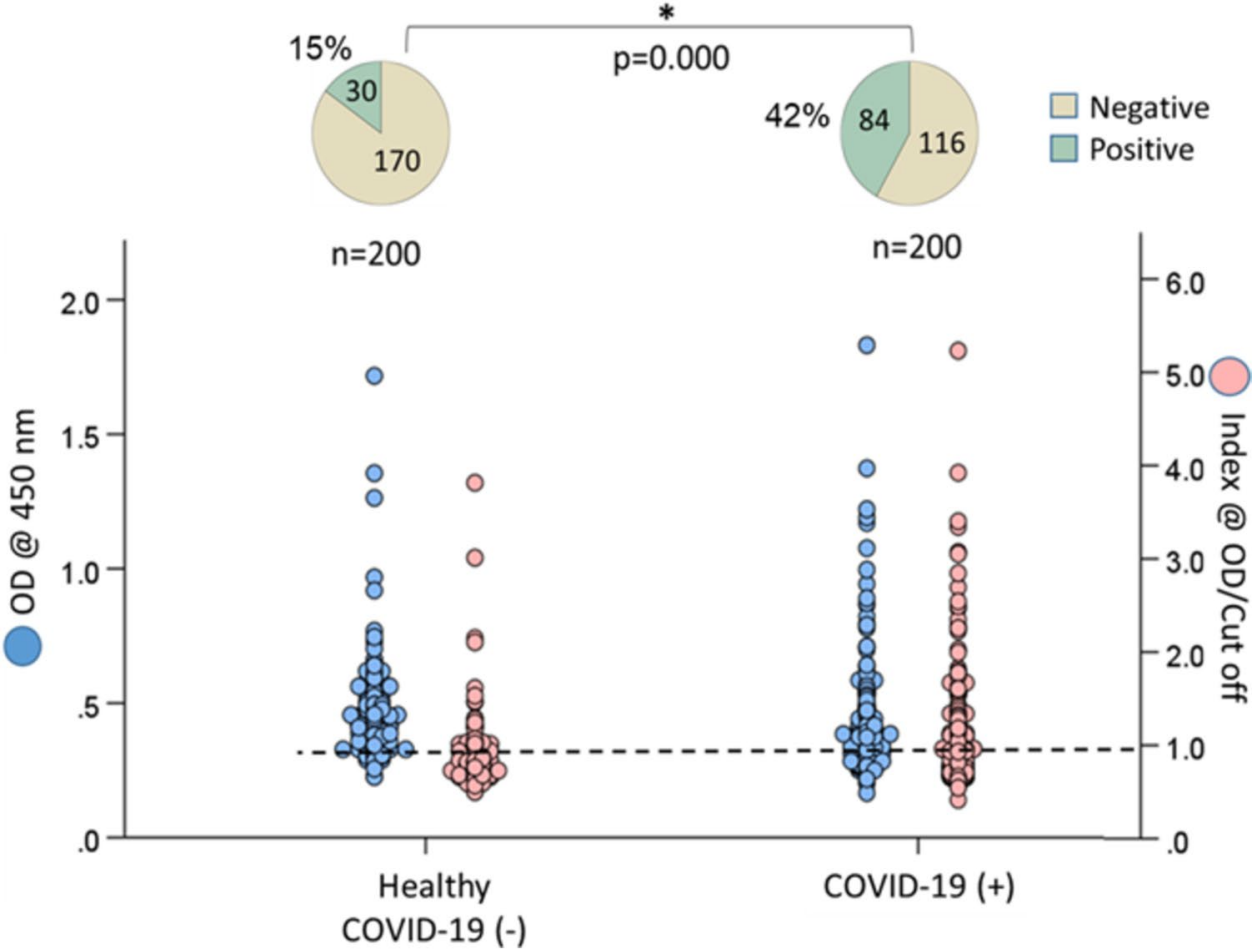
The rates of anti-Hep-2 nucleus antibody positivity in individuals who have recovered from COVID-19 compared to healthy controls

Among the 200 healthy individuals, 38 (19%) tested positive for anti-dsDNA, 37 (18.5%) for anti-ENA, and 30 (15%) for anti-Hep-2 nucleus antibodies. In contrast, the rates of ANA positivity significantly increased in the COVID-19 group, with 97 individuals (48.5%) testing positive for dsDNA, 81 (40.5%) for ENA, and 84 (42%) for Hep-2 nucleus antibodies (*p* < 0.05) (Figs. 2, 3, and 4).

### Distribution of ANA positivity by gender

When evaluating the dsDNA, ENA, and Hep-2 nucleus tests collectively, it was found that two out of every three ANA-positive individuals, both among healthy individuals and those who had COVID-19, were women (Table 1) (*p* < 0.05).

**Table 1:** Distribution of ANA positivity in healthy individuals and those with COVID-19 by gender

	Healthy			COVID-19 ( +)		
	Man	Woman	p	Man	Woman	*p*
dsDNA	13 (34%)	25 (66%)	0.006	32 (33%)	65 (67%)	0.000
ENA	9 (24%)	28 (76%)	0.000	30 (37%)	51 (63%)	0.001
Hep-2 nucleus	6 (20%)	24 (80%)	0.000	26 (31%)	58 (69%)	0.000

## Discussion

### Increased prevalence of ANA in the post-COVID-19 period

The ANA positivity rate was found to have increased by over 100% across all three types of ANA tests (dsDNA, ENA, and Hep-2 nucleus) compared to healthy individuals who did not contract COVID-19 (see Figs. 2, 3, and 4). Previous studies with smaller sample sizes (*n* = 33–131) identified ANA as the most prevalent type of autoantibody in COVID-19 patients [12–16]. Consistent with the findings of the current study, these investigations revealed that ANA prevalence increased two to threefold relative to healthy controls. For instance, Paskolini et al. reported positive ANA tests in 11 out of 33 COVID-19 cases (33%) [14]. Similarly, Vahabi et al. examined 131 COVID-19 patients and found a positivity rate of 36.4% (48/131) for ANA [13]. Woodruff et al. investigated 52 adult COVID-19 patients from diverse racial backgrounds and found that 44% developed ANA [15]. These findings suggest that the relationship between COVID-19 and ANA positivity is independent of geographic and racial factors.

Furthermore, when evaluated alongside other similar studies in the literature, the current study’s large sample size supports the conclusion that COVID-19 may trigger autoimmune reactions [17].

### Explanations for increased ANA rates following COVID-19

Several potential reasons may account for the observed increase in ANA rates following COVID-19. A significant factor is the widespread and prolonged inflammation associated with the disease, which can lead to hypersensitivity in the immune system [18]. This hypersensitivity is believed to trigger autoantibody production through abnormal immune responses, such as cytokine storms or the overactivation of B lymphocytes [19, 20]. Liu et al. (2021) suggested that excessive immune activity in COVID-19 disrupts immune tolerance, increasing the likelihood of cross-reactivity with host cells, thereby resulting in autoantibody formation [20]. Similarly, Garmendia et al. (2022) noted a potential link between the production of anti-interferon autoantibodies and B cell overactivation [21].

Another hypothesis posits that viral epitopes may share similarities with self-antigens, leading antibodies produced against the virus to cross-react with self-structures and trigger autoantibody production [22]. Research has identified 14 epitopes in tissues such as the thyroid, pituitary gland, adrenal cortex, and islet beta cells that exhibit similarities to the SARS-CoV-2 S-protein [23, 24]. This epitope similarity may explain the various autoimmune disorders observed in COVID-19 patients, potentially resulting in organ damage. For example, Churilov et al. [22] demonstrated that autoantibodies targeting the adrenal glands in COVID-19 could impair adrenal function, crucial for an adequate response to cytokine storms [25]. The failure of the adrenal response due to these autoantibodies can lead to an excessive release of inflammatory mediators, exacerbating the cytokine storm [25]. Rathmann et al. (2022) reported that autoantibodies against the islets of Langerhans contribute to the development of Type II diabetes following COVID-19 [26]. Moreover, the molecular similarity theory has implications not only for COVID-19 but also for various other viral infections, such as adenovirus, CMV, EBV, HBV, and HIV, implicating autoantibodies in numerous disorders arising in these patients [27].

Another theory suggests that viral infections like COVID-19 can induce changes in self-antigens, triggering autoimmunity. It has been observed that oxidants, produced in increased quantities during infection, may modify type 2 collagen, leading to qualitative changes in self-antigens that stimulate autoimmune responses [28]. This mechanism is thought to underlie the formation of anti-CCP antibodies observed in COVID-19 cases [29]. Additionally, the extensive involvement of disease in COVID-19 may delay the clearance of apoptotic cells, subsequently promoting the formation of autoantibodies against intracellular components, such as ANAs [30]. Thus, the risk of autoantibody formation is reported to increase with the duration of COVID-19 illness [8, 9]. In conclusion, the findings of the current study, in conjunction with existing literature, suggest that SARS-CoV-2 may function as an autoimmune virus [21, 31].

### Increased ANA positivity rate in healthy individuals

An important finding in our study is the elevated ANA positivity rate among healthy individuals, which was higher than the literature average. While existing literature indicates that ANA positivity occurs in 2–15% of healthy individuals, this rate has risen to 15–20% in Türkiye. Dinse et al. [31] examined the prevalence of ANA over three distinct periods (1988–1991, 1999–2004, and 2011–2012) and reported a progressive increase in ANA positivity rates (11%, 11.5%, and 15.9%, respectively) (33). Given the lack of comprehensive screenings for ANA prevalence in healthy individuals since 2020, it is plausible that this rate has approached 20% in recent years. The data from the current study, representing the latest investigation into ANA prevalence, corroborate this upward trend. Considering that ANA positivity may serve as an indicator of rising autoimmune diseases, further investigation into the reasons behind this elevated rate among healthy individuals is warranted.

## Conclusion

The SARS-CoV-2 virus significantly increases the formation of antinuclear antibodies. Given that a large portion of the population has been exposed to this virus and its variants, an increase in the prevalence of autoimmunity within the community may be anticipated. Furthermore, the prevalence of ANA positivity in healthy individuals approaching 20% independent of COVID-19 is an important finding that warrants further investigation.

### Strengths and limitations of the study

One of the strengths of the study is that it was conducted with a larger sample size (*n* = 400) compared to previous studies in the literature. Another strength is the use of the Hep-2 cell nuclear test alongside the commonly used dsDNA and ENA tests for ANA measurements. This test, which utilizes the Hep-2 nucleus rich in ANA antigens, was crucial for detecting ANA-positive samples that may not be identified by the dsDNA and ENA tests.

A limitation of the study is that in individuals with positive ANA, the potential role of ANA in contributing to autoimmune disorders could not be evaluated due to the retrospective design. Additionally, other types of autoantibodies were not examined. However, the high prevalence of ANA in individuals who have had COVID-19 can still be considered significant evidence for the risk of autoimmunity.

## Data Availability

The data that support the findings of this study are available from the corresponding author upon reasonable request.
